# Selecting normalization genes for small diagnostic microarrays

**DOI:** 10.1186/1471-2105-7-388

**Published:** 2006-08-22

**Authors:** Jochen Jaeger, Rainer Spang

**Affiliations:** 1 Max Planck Institute for Molecular Genetics, Ihnestrasse 73, 14195 Berlin, Germany

## Abstract

**Background:**

Normalization of gene expression microarrays carrying thousands of genes is based on assumptions that do not hold for diagnostic microarrays carrying only few genes. Thus, applying standard microarray normalization strategies to diagnostic microarrays causes new normalization problems.

**Results:**

In this paper we point out the differences of normalizing large microarrays and small diagnostic microarrays. We suggest to include additional normalization genes on the small diagnostic microarrays and propose two strategies for selecting them from genomewide microarray studies. The first is a data driven univariate selection of normalization genes. The second is multivariate and based on finding a balanced diagnostic signature. Finally, we compare both methods to standard normalization protocols known from large microarrays.

**Conclusion:**

Not including additional genes for normalization on small microarrays leads to a loss of diagnostic information. Using house keeping genes from the literature for normalization fails to work for certain datasets. While a data driven selection of additional normalization genes works well, the best results were obtained using a balanced signature.

## Background

Several publications have suggested the use of cDNA-microarrays for clinical diagnosis [1-4]. While today's microarrays can cover up to 50,000 genes, only a small percentage of them is needed for diagnosis. Most diagnostic microarray datasets can achieve optimal classification with no more than 5–50 discriminative genes [5-7]. This opens new possibilities for the design of small diagnostic microarrays used for gene expression based diagnosis.

To design such disease specific, small custom arrays differential genes are identified from a large set of potential candidate genes using genome wide expression profiling. Then, only these differential genes are put onto a small custom microarray [8]. Throughout this paper, we refer to diagnostic microarrays as small, custom microarrays for diagnostic purpose holding only few genes and large microarrays as genomewide gene expression microarrays, holding tens of thousands of genes.

With the concept of diagnostic microarrays new problems arise. A first important step in microarray analysis is normalization. The overall intensity of microarrays can vary in a large dataset. This can reflect global differential gene expression, but it is more likely due to experimental artifacts. Consequently, array-to-array normalization is crucial for microarray analysis [9-11]. Various methods for normalization have been suggested. One approach is to determine a set of invariant genes for normalization [12,13]. Another approach recommends to replicate genes on the array and use this within-array replication for normalization [8,14,15].

Standard normalization protocols rely on the assumption that the majority of genes on the microarray are not differentially expressed between samples [9]. For large microarrays this is likely to be true, but on a diagnostic microarray the genes are selected to be differentially expressed between disease entities. Consequently, for these diagnostic microarrays a fundamental assumption of microarray normalization does not hold. This has negative effects on the quality of gene expression measurements. Assume that a diagnostic signature consists of 10 genes, all of them higher expressed in disease type A than in type B. Since there are also scale differences due to experimental artifacts, the microarrays need to be normalized. Normalizing them to constant average expression also eliminates the biological differences between A and B. The dilemma is that global differences can be either artifacts or the manifestation of molecular difference between the disease types. Diagnostic microarrays need to be designed in a way that allows for the discrimination of these two different effects.

One way to address this problem is to include additional genes on the microarray that are exclusively used for normalization. Typically, one uses housekeeping genes, which are thought to be expressed at a constant level. However, it has been found that housekeeping genes are occasionally regulated, too [16-18].

Therefore, we suggest a data driven approach to select normalization genes from the pool of all genes on the microarray. Not only the diagnostic signature should be derived from the analysis of a large microarray study but we suggest that this data is also used for finding normalization genes. These are then used for normalizing the diagnostic microarrays. Note that there is a conceptual difference between choosing an invariant set of genes from the data you want to normalize [12,13], and selecting genes from one dataset (a genome wide expression study) for the purpose of normalizing a second dataset (a diagnostic array). In the first scenario the variance of genes does not need to generalize to new data. In the second scenario it does.

Here we address the problem of selecting normalization genes from a genome wide expression study for the purpose of designing diagnostic arrays. The goal is that the diagnostic array can then be normalized without problems. We compare two simple strategies in the context of simulation experiments as well as in real world applications. The first strategy aims to find control genes that are not influenced by the disease type and can therefore be used for normalization. The second strategy aims to find genes that complement the discriminatory genes on the diagnostic microarray in a way such that a normalization function on all genes together is not any more influenced by the diseases type. We call this novel concept *balanced signatures*.

The paper is organized as follows: First we demonstrate the problems occurring when standard normalization protocols are used for diagnostic microarrays. In the "Methods" section we discuss alternative strategies for normalization gene selection and the concept of balanced signatures. In the "Results and Discussion" section we compare our methods in the setting of a controlled simulation experiment. In the "Results on lung cancer and leukemia studies" section we show normalization performance on a dataset from a clinical study on leukemia and on a dataset from a clinical study on lung cancer. We close with a summary and a discussion of our findings.

## Results and discussion

### Simulated data

The two normalization methods for diagnostic microarrays described in the "Methods" section need to be evaluated with respect to their power in compensating the global signal normalization effect and producing diagnostic arrays that distinguish well between two disease entities. Before we evaluate our methods on real data in the next section we make use of the more transparent setting of a simulation study, in which the population differences, the biological variability among individuals and the experimental variability are modeled independently of each other.

Simulated data was generated according to a multivariate normal distribution, including strong correlation of genes, a large spectrum of expression intensities and non constant expression differences between the two groups A and B.

In total we simulated expression values for 3000 genes on 50 microarrays representing two groups A and B of 25 microarrays each. We first generated the covariance matrix Σ by randomly drawing from an inverse Wishart distribution with 3150 degrees of freedom and a 3000 × 3000 identity matrix as a scale matrix. Then, we generated a vector of 3000 population means for each gene *i *= {1,..., 3000} in each group *A *and *B*, μiA
 MathType@MTEF@5@5@+=feaafiart1ev1aaatCvAUfKttLearuWrP9MDH5MBPbIqV92AaeXatLxBI9gBaebbnrfifHhDYfgasaacH8akY=wiFfYdH8Gipec8Eeeu0xXdbba9frFj0=OqFfea0dXdd9vqai=hGuQ8kuc9pgc9s8qqaq=dirpe0xb9q8qiLsFr0=vr0=vr0dc8meaabaqaciaacaGaaeqabaqabeGadaaakeaaiiGacqWF8oqBdaqhaaWcbaGaemyAaKgabaGaemyqaeeaaaaa@30FC@, μiB
 MathType@MTEF@5@5@+=feaafiart1ev1aaatCvAUfKttLearuWrP9MDH5MBPbIqV92AaeXatLxBI9gBaebbnrfifHhDYfgasaacH8akY=wiFfYdH8Gipec8Eeeu0xXdbba9frFj0=OqFfea0dXdd9vqai=hGuQ8kuc9pgc9s8qqaq=dirpe0xb9q8qiLsFr0=vr0=vr0dc8meaabaqaciaacaGaaeqabaqabeGadaaakeaaiiGacqWF8oqBdaqhaaWcbaGaemyAaKgabaGaemOqaieaaaaa@30FE@ by randomly drawing from a *N *(0,1) normal distribution for each gene and group. The actual expression data was generated by drawing from a multivariate normal distribution with covariance matrix Σ and means *μ*_*A *_for the first 25 microarrays and *μ*_*B *_for the next 25 microarrays. Finally, this data was perturbed by multiplying with a random scaling factor and adding a random offset both drawn from a N(0,0.3) log-normal distribution. The generation of this data was done twice. Once for a training set and once for a test set.

In this simulation with three successive randomization steps the first step of generating *μ*^*A*^, *μ*^*B *^and Σ corresponds to the population properties of the genes. The second step of drawing from a multivariate *N*(*μ*^*A*,*B*^, Σ) distribution accounts for biological variability among individuals, while the third step of perturbing the data accounts for global experimental artifacts. The observed differences Δ_*i *_display the typical continuous spectrum known from real expression data (figure 2).

As we have stressed before, the expression patterns of the normalization genes need to generalize from the training set where they were found to new data in the same way as the signature patterns do. From the theoretical considerations of the "Methods" section it becomes clear that small variance genes have the potential to compensate for the global signal normalization effect. But the genes need to have small variances not only on the training data but also and more importantly on the data that is generated using the diagnostic array. In general, this variance will be higher than it is on the training data. The same problem occurs for genes with small average expression differences and balanced signatures. To this end, we simulated a training and a test set with 50 samples. Both sets have the same underlying gene means and covariance structure. To avoid overfitting, only the training data was used to select the normalization genes and only the test set was used to evaluate the normalization strategies. The diagnostic signature consists of *p*_*s *_= 10 genes with the largest difference of population means. It is unbalanced. For the purpose of normalization *p*_*n *_= 10 additional genes were picked according to the suggested methods.

Using the standard normalization protocol destroys the signal completely, while using random normalization genes already recovers the signal partially (left plot in figure 3). However, both versions, data based selection of normalization genes and balanced signatures, recover population differences more accurately and perform similarly to each other (right plot in figure 3).

We repeated the data simulation 30 times and recorded for each simulation the distance between the real underlying expression differences of the signature genes and the expression differences obtained by the various normalization methods. This sum of squared error plot shows that all methods achieve significantly better normalization results compared to the standard method (*p *< 10^-7 ^using a paired Wilcoxon test). The balanced signatures also perform better than the other proposed methods (figure 4). In the case of "small effect normalization", "small CV", and "random" this difference is significant (*p *< 0.012), while in the case of "variance normalization" significance on the 0.05 level was not achieved (*p *= 0.17).

#### Two exemplary clinical studies

We now proceed from a simulation study to applications on real datasets. Of course, in real datasets we do not know how many genes are deregulated and how many are necessary for achieving optimal classification accuracy. Therefore, we ran the *MCRestimate *package [20], that uses a nested cross validation loop to avoid biased estimators of classification performance. Our own results analyzing various datasets with *MCRestimate *showed that most datasets can be classified optimally with a handful of genes and only very few need more than 50 (data not shown). This is in concordance with findings from other authors [5-7]. When applying it to the leukemia study [2], described in the "Methods" section, we found that in this case *p*_*s *_= 5 genes reached the optimal classification accuracy of 99%. Thus, we selected *p*_*s *_= 5 signature genes with the highest absolute equal variance t-score. In addition, *p*_*n *_= 5 normalization genes were determined according to the criteria from the "Methods" section. For simplicity, the number *p*_*n *_of additional genes for normalization was set to *p*_*s*_. In preliminary studies this provided good results but further research on determining the optimal *p*_*s *_and *p*_*n *_simultaneously is needed.

The second dataset we analyzed was a study on 86 primary lung adenocarcinoma and 10 normal lung tissues [21]. Here, we aimed for a classification of normal versus carcinoma. *MCRestimate *achieved 100% accuracy using 3 genes. Thus, we selected *p*_*s *_= *p*_*n *_= 3 for this dataset.

We randomly split the whole datasets equally into a training and test set. For the training set we applied the gold standard normalization using all genes of the large microarray. Then, we proceeded in the same way as described in the "Methods" section. Both, signature and normalization genes were derived using only the training data. For each sample in the test set a diagnostic microarray was constructed using only the raw data of the signature and the normalization genes. This diagnostic microarray was normalized using the procedure described in the "Methods" section, resulting in seven different test datasets: standard protocol, Affymetrix housekeeping genes, random normalization genes, low variances, small coefficient of variation, small differences and balanced signatures. On the such normalized test set we evaluated the normalization methods with respect to the diagnostic performance of a support vector machine using cross validation. For this, we used the SVM from the package *e1071 *in R [22] with a linear kernel and default parameters. The dataset was randomly split in equally sized training and test sets. This was repeated 100 times and the evaluation steps were rerun for every data partitioning.

The standard protocol reduces the classification accuracy substantially, while both normalization gene selection and balanced signatures yield satisfying results. Affymetrix housekeeping genes for normalization work well on the leukemia dataset, but fail on the lung dataset. Balanced signatures provide the best results in both datasets.

For the leukemia dataset classification accuracy was significantly better for all our methods as compared to the standard protocol (*p *< 10^-15^). "Balanced normalization" outperformed all other normalizations (*p *< 10^-8^), too. Standard normalization was also clearly inferior in the lung dataset (*p *< 10^-14^). When further testing "balanced normalization" against other normalizations p-values were below 0.001 for all but "small effect normalization" and "random normalization", where significance was not reached (*p *= 0.13 and *p *= 0.15 respectively).

## Conclusion

In this paper we showed that using a standard normalization protocol from large microarrays has fatal effects. They are most pronounced when the diagnostic signature is unbalanced, containing more up- than down-regulated genes or vice versa. However, in most microarray datasets there are more significantly up- than down-regulated genes or vice versa, emphasizing the need for new normalization strategies. Here, we introduced two strategies to overcome this problem: normalization gene selection and balanced signatures. Both gave better results for diagnostic microarrays than the standard normalization protocol. Using Affymetrix housekeeping genes performs well in the analyzed leukemia dataset but does not work for the lung dataset, indicating that these genes are actively regulated in these tissues.

As standard normalization protocol we have chosen the RMA procedure. Of course it is not the only protocol in use. However, the global signal normalization effect is generic and not restricted to this protocol. Any normalization which assumes unchanged expression for the majority of genes on the microarray is expected to suffer from the same problem. An advantage of both our methods is that the normalization genes can be selected with no additional experimental cost and little computational effort.

In recent publications it was shown that the list of differentially expressed genes are unstable and the overlap of gene lists from different analysis is small [23-25]. However, for diagnosis one is not aiming at finding a unique set of signature genes, but a unique diagnosis of future patients. There are many datasets containing man different sets of genes, which all lead to the same diagnosis. For the purpose of designing diagnostic arrays it is sufficient to find one such set.

Hua et al. stressed that optimal feature size depends strongly on the classifier and feature-label distribution and that a choice of optimal feature size can greatly improve accuracy of the classification [26]. Hence, for assessing how many genes should be used for a diagnostic microarray we used a nested cross validation for SVMs [20]. By this, we determined the number of genes making up the diagnostic signature (*p*_*s*_) and set it to the number of genes needed for achieving the optimal classification accuracy.

In conclusion, balanced signatures perform well with respect to recovering the real underlying signal as well as for classification. This was verified on a simulated test dataset as well as on two real microarray datasets. Their main advantage is that no space on the diagnostic microarray is wasted and all genes can be integrated in the diagnostic signature.

## Methods

Standard microarray normalization protocols can not be directly applied to diagnostic microarrays because ignoring the special character of normalization on diagnostic microarrays leads to a loss of the biological signal. To illustrate this normalization effect on real data, we used a publicly available dataset on acute lymphocytic leukemia (ALL) in children [2]. It consists of 327 samples that fall into different clinical classes characterized by immunophenotype, chromosomal translocations and aberrations. The study was carried out using Affymetrix HGU95Av2 chips with 12625 probesets covering more than 9000 known human genes. For these large Affymetrix chips we applied a standard normalization protocol where we preprocessed the data using background correction followed by probeset summarization and finally normalization on the summary values. Background correction was done using perfect match (PM) probes only, ignoring mismatch (MM) probes. Probeset summary was done using an additive model fitted by a median polish procedure. Finally, the data was quantile normalized. We used the *RMA *package [19] with default parameters to perform all three steps. Note that the probeset summarization step takes logarithms of the data and hence transforms expression levels to an additive scale. Here, fold changes of molecule abundance correspond to differences in the normalized data.

We now mimic a potential diagnostic microarray for discriminating between patients displaying a TEL-AML translocation (group A) and those displaying either a BCR-ABL or a E2A-PBX1 translocation (group B). To this end, we discard all data except for the set of genes that is selected for a diagnostic array. This set includes signature genes and additional normalization genes. Of course, this diagnostic microarray was not physically built but constructed in the computer. Nevertheless, it still consists of real data. More precisely, we chose the 10 most upregulated genes in group A. To mimic a diagnostic array we went back to the non-normalized raw data of only these 10 genes and discarded all other expression data. Using only the remaining raw data of these 10 genes we repeated the same normalization steps that were used for the large Affymetrix microarray. Since normalization was not done on an array-by-array, nor on a gene-by-gene basis, but borrowed information across both genes and microarrays the results of the two normalizations were different although the underlying raw data was identical.

**Figure 1:**
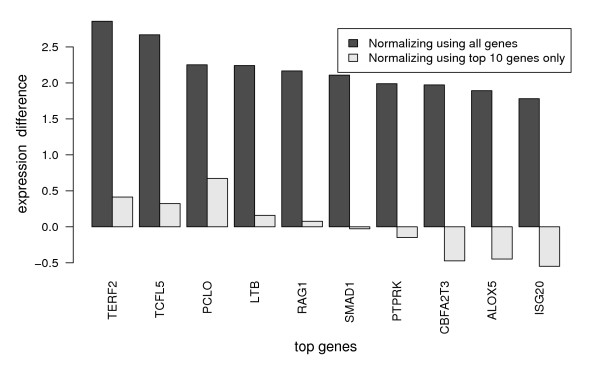
**Normalization effect on diagnostic microarrays**. The global signal normalization effect resulting from standard normalization protocols applied to diagnostic microarrays: Shown are changes of expression difference, when switching from a large microarray to a diagnostic microarray. The top genes are those genes with the maximal expression difference between TEL-AML versus BCR-ABL and E2A-PBX1. Note, that expression differences on log scale reflect fold changes.

**Figure 2:**
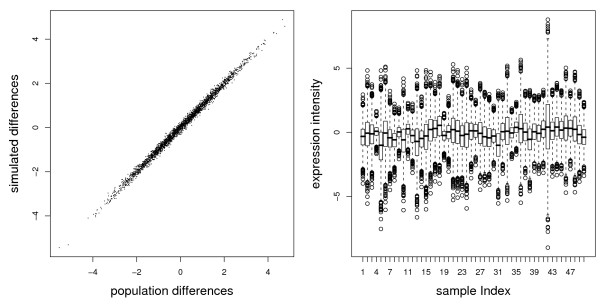
**Characteristics of simulated data**. The left plot shows the genewise population differences contrasted with the mean differences in simulated data. Population differences μiA
 MathType@MTEF@5@5@+=feaafiart1ev1aaatCvAUfKttLearuWrP9MDH5MBPbIqV92AaeXatLxBI9gBaebbnrfifHhDYfgasaacH8akY=wiFfYdH8Gipec8Eeeu0xXdbba9frFj0=OqFfea0dXdd9vqai=hGuQ8kuc9pgc9s8qqaq=dirpe0xb9q8qiLsFr0=vr0=vr0dc8meaabaqaciaacaGaaeqabaqabeGadaaakeaaiiGacqWF8oqBdaqhaaWcbaGaemyAaKgabaGaemyqaeeaaaaa@30FC@ - μiB
 MathType@MTEF@5@5@+=feaafiart1ev1aaatCvAUfKttLearuWrP9MDH5MBPbIqV92AaeXatLxBI9gBaebbnrfifHhDYfgasaacH8akY=wiFfYdH8Gipec8Eeeu0xXdbba9frFj0=OqFfea0dXdd9vqai=hGuQ8kuc9pgc9s8qqaq=dirpe0xb9q8qiLsFr0=vr0=vr0dc8meaabaqaciaacaGaaeqabaqabeGadaaakeaaiiGacqWF8oqBdaqhaaWcbaGaemyAaKgabaGaemOqaieaaaaa@30FE@ were set for each gene by randomly drawing from N(0,1). Simulated differences stem from drawing data from a multivariate distribution with these given population means. The right plot shows boxplots of all 3000 genes for all 50 samples of the simulated data for the training set (the test set is very similar and not shown).

**Figure 3:**
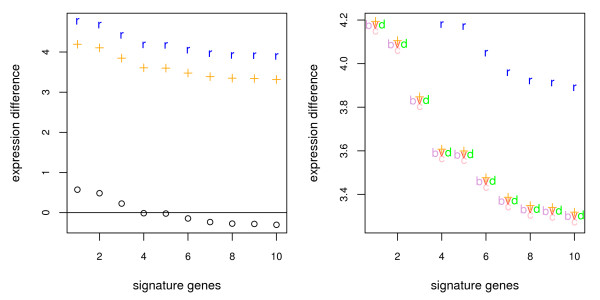
**Recovery of original effect using different normalization methods**. Effects of different normalization methods for diagnostic microarrays evaluated on simulated data. " + " depicts expression differences in the test data of the signature genes after normalization with all 3000 genes. This, we would like to recover with normalization methods for diagnostic microarrays, too."o" corresponds to using the standard protocol on the diagnostic microarray. Here, all the signal is lost, "r" corresponds to a normalization of the diagnostic microarray with 10 random genes. It already recovers the signal partially. The right plot is a closeup of the left plot, showing additionally the performance of the proposed normalization schemes. " + " and "r" are the same as in the left plot. Additionally, normalization using lowest variance "v", smallest difference "d", smallest coefficient of variation "c" and balanced signatures "b" are shown. For better visibility the symbols "b" and "d" are slightly moved to the side so that they do not overlap.

**Figure 4:**
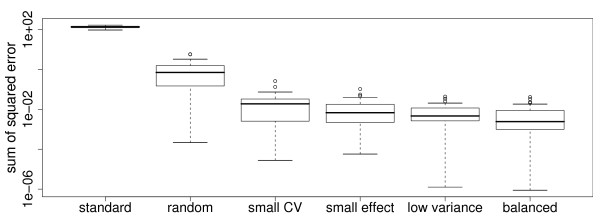
**Loss of effect for different normalization methods**. Sum of squared errors to the real underlying expression differences of the proposed normalization methods and the standard protocol averaged over 30 runs of the simulated data. "Small CV" depicts the normalization method using smallest coefficient of variation and "small effect" depicts the normalization method using small differences of average expression.

When switching from the large microarray to the diagnostic microarray the expression differences between the two cytogenetically different groups of patients vanished almost completely. Normalization of the diagnostic microarray had destroyed the original signal needed for diagnosis (Figure 1). We refer to this effect as the *global signal normalization effect*. Not only did the expression differences vanish, but the average correlation between the genes also changed from 0.73 to -0.1.

We showed that standard normalization applied to diagnostic microarrays can substantially skew results and is a problem for diagnosis. In the following section we propose two different strategies to circumvent these problems. The first strategy aims at finding genes that can be used solely for normalization. Several methods for finding these genes are suggested and compared. The second strategy aims at finding genes that can be used for normalization and additionally also for classification.

### Diagnostic microarray normalization with selected genes

We have argued that a microarray carrying only differentially expressed genes can hardly be used to distinguish biological effects from experimental artifacts. To overcome this problem we suggest to include additional normalization genes on a diagnostic microarray that are then used to adjust for experimental artifacts but leave the biological signal intact. Like the signature genes, the normalization genes can be selected based on the data from a genomewide expression study. While signature genes should correlate with the disease labels of patients, the normalization genes should not.

For the signature genes it is most important that the correlation of expression levels to the disease labels does not only hold for the training data on which the genes were found but generalizes to new samples. In the same way the desired properties of normalization genes also need to generalize to new data. Hence, criteria for normalization need to be chosen such that they enable both, a good normalization of diagnostic microarrays and at the same time generalize well to new samples. Note that these two requirements do not implicate each other.

Let *p*_*s *_be the number of genes that form the diagnostic signature. In experimental settings *p*_*s *_was in the range of 5–50 genes [5-7]. Let *p*_*n *_be the number of additional genes used on the microarray for array-to-array normalization. The total number of genes on the diagnostic microarray is thus *p*_*d *_= *p*_*s *_+ *p*_*n*_. Both the signature genes and the normalization genes are selected based on genomewide microarray data measured with large microarrays holding *p*_*l *_» *p*_*d *_genes. In this context *x*_*ij *_denotes the expression of gene *i *in patient *j*. As we aim at diagnostic differentiation into groups we can assume without loss of generality that the samples fall into two different disease entities represented by class labels A and B. If there should be more classes, it is always possible to construct a binary classification tree where the first group is compared to all others. Then the second group is compared to the rest excluding the first group and so on.

The open question is how to select normalization genes. We propose two novel methods. The first method selects genes solely used for normalization according to criteria listed below. The second method aims at balancing the signature and is described in the section "Balanced signatures".

### Selection of normalization genes

For the first method we suggest three alternative criteria:

#### 1. Low variance genes

Calculate the empirical variance σi2
 MathType@MTEF@5@5@+=feaafiart1ev1aaatCvAUfKttLearuWrP9MDH5MBPbIqV92AaeXatLxBI9gBaebbnrfifHhDYfgasaacH8akY=wiFfYdH8Gipec8Eeeu0xXdbba9frFj0=OqFfea0dXdd9vqai=hGuQ8kuc9pgc9s8qqaq=dirpe0xb9q8qiLsFr0=vr0=vr0dc8meaabaqaciaacaGaaeqabaqabeGadaaakeaaiiGacqWFdpWCdaqhaaWcbaGaemyAaKgabaGaeGOmaidaaaaa@30F0@ of all *p*_*l *_genes and choose the *p*_*n *_genes with the smallest variance in the data. Use only these genes for array-to-array normalization. In our preprocessing protocol the background correction and probeset summarization remain unchanged but only these *p*_*n *_genes are used for the final normalization step.

In this approach, we aim for the genes with the most constant expression in both disease populations. Population variances are not known and we select the genes due to their variances on the expression data of the genomewide study. This idea is similar to the use of housekeeping genes, whose expression is assumed to hardly vary between patients. Observed differences in measurements are hence most likely due to experimental artifacts. However, we do not select housekeeping genes based on a priori knowledge, but from the data at hand.

#### 2. Small coefficient of variation

Calculate the empirical variance σi2
 MathType@MTEF@5@5@+=feaafiart1ev1aaatCvAUfKttLearuWrP9MDH5MBPbIqV92AaeXatLxBI9gBaebbnrfifHhDYfgasaacH8akY=wiFfYdH8Gipec8Eeeu0xXdbba9frFj0=OqFfea0dXdd9vqai=hGuQ8kuc9pgc9s8qqaq=dirpe0xb9q8qiLsFr0=vr0=vr0dc8meaabaqaciaacaGaaeqabaqabeGadaaakeaaiiGacqWFdpWCdaqhaaWcbaGaemyAaKgabaGaeGOmaidaaaaa@30F0@ and the empirical mean *μ*_*i *_of all *p*_*l *_genes and choose the *p*_*n *_genes with the smallest coefficient of variation σiμi
 MathType@MTEF@5@5@+=feaafiart1ev1aaatCvAUfKttLearuWrP9MDH5MBPbIqV92AaeXatLxBI9gBaebbnrfifHhDYfgasaacH8akY=wiFfYdH8Gipec8Eeeu0xXdbba9frFj0=OqFfea0dXdd9vqai=hGuQ8kuc9pgc9s8qqaq=dirpe0xb9q8qiLsFr0=vr0=vr0dc8meaabaqaciaacaGaaeqabaqabeGadaaakeaadaWcaaqaaGGaciab=n8aZnaaBaaaleaacqWGPbqAaeqaaaGcbaGae8hVd02aaSbaaSqaaiabdMgaPbqabaaaaaaa@334F@ in the data. Use only these *p*_*n *_genes for array-to-array normalization.

In this approach, we aim for the genes with low variance that additionally have high intensity. The idea is to exclude low variance genes within the background noise.

#### 3. Small differences of average expression

Calculate the differences Δ_*i *_= ∑_*j *∈ *JA *_*x*_*ij*_/|*J*_*A*_|-∑_*j *∈ *JB *_*x*_*ij*_/|*J*_*B*_| between the two groups for all *p*_*l *_genes and choose the *p*_*n *_genes with the smallest absolute Δ_*i*_. Use only these genes for array-to-array normalization.

In this approach we allow the genes to vary between patients but this variability should not correlate with the disease type. Note that the genes are typically not constant and therefore not housekeeping genes. Still they allow for normalization if the property of small expression differences generalizes well to the diagnostic microarray.

As a control we used randomly sampled genes for normalization. Here of course we have no problem with generalization. One might expect, that the above methods are more effective, but this needs to be proved empirically.

For the evaluation of the real datasets we included the normalization results obtained when using standard housekeeping genes. For this, we used the following 3' variants of the housekeeping probe-sets supplied on Affymetrix GeneChips: beta-actin, GAPDH, ISGF3, 18S rRNA, transferrin receptor and 28S rRNA.

### Balanced signatures

This approach does not use different genes for normalization and diagnosis, but tries to find a set of genes, which serves both tasks at the same time. Starting from a non balanced set of signature genes, choose *p*_*n *_genes from all *p*_*l *_genes such that the variation of the average gene expression per microarray is minimized

∑j∈J(x.j−x..)2→min⁡⇒∑j∈J(∑i∈Idxij|Id|−∑i∈Id∑j∈Jxij|Id|∗|J|)2→min⁡⇒∑j∈J(∑i∈Id(xij−∑j∈Jxij|J|))2→min⁡
 MathType@MTEF@5@5@+=feaafiart1ev1aaatCvAUfKttLearuWrP9MDH5MBPbIqV92AaeXatLxBI9gBaebbnrfifHhDYfgasaacH8akY=wiFfYdH8Gipec8Eeeu0xXdbba9frFj0=OqFfea0dXdd9vqai=hGuQ8kuc9pgc9s8qqaq=dirpe0xb9q8qiLsFr0=vr0=vr0dc8meaabaqaciaacaGaaeqabaqabeGadaaakeaafaqabeWabaaabaWaaabuaeaadaqadiqaaiabdIha4jabc6caUmaaBaaaleaacqWGQbGAaeqaaOGaeyOeI0IaemiEaGNaeiOla4IaeiOla4cacaGLOaGaayzkaaWaaWbaaSqabeaacqaIYaGmaaaabaGaemOAaOMaeyicI4SaemOsaOeabeqdcqGHris5aOGaeyOKH4QagiyBa0MaeiyAaKMaeiOBa4MaeyO0H4nabaWaaabuaeaadaqadiqaamaaqafabaWaaSaaaeaacqWG4baEdaWgaaWcbaGaemyAaKMaemOAaOgabeaaaOqaamaaemGabaGaemysaK0aaSbaaSqaaiabdsgaKbqabaaakiaawEa7caGLiWoaaaGaeyOeI0YaaabuaeaadaaeqbqaamaalaaabaGaemiEaG3aaSbaaSqaaiabdMgaPjabdQgaQbqabaaakeaadaabdiqaaiabdMeajnaaBaaaleaacqWGKbazaeqaaaGccaGLhWUaayjcSdGaey4fIOYaaqWaceaacqWGkbGsaiaawEa7caGLiWoaaaaaleaacqWGQbGAcqGHiiIZcqWGkbGsaeqaniabggHiLdaaleaacqWGPbqAcqGHiiIZcqWGjbqsdaWgaaadbaGaemizaqgabeaaaSqab0GaeyyeIuoaaSqaaiabdMgaPjabgIGiolabdMeajnaaBaaameaacqWGKbazaeqaaaWcbeqdcqGHris5aaGccaGLOaGaayzkaaWaaWbaaSqabeaacqaIYaGmaaGccqGHsgIRcyGGTbqBcqGGPbqAcqGGUbGBcqGHshI3aSqaaiabdQgaQjabgIGiolabdQeakbqab0GaeyyeIuoaaOqaamaaqafabaWaaeWaceaadaaeqbqaamaabmGabaGaemiEaG3aaSbaaSqaaiabdMgaPjabdQgaQbqabaGccqGHsisldaaeqbqaamaalaaabaGaemiEaG3aaSbaaSqaaiabdMgaPjabdQgaQbqabaaakeaadaabdiqaaiabdQeakbGaay5bSlaawIa7aaaaaSqaaiabdQgaQjabgIGiolabdQeakbqab0GaeyyeIuoaaOGaayjkaiaawMcaaaWcbaGaemyAaKMaeyicI4SaemysaK0aaSbaaWqaaiabdsgaKbqabaaaleqaniabggHiLdaakiaawIcacaGLPaaaaSqaaiabdQgaQjabgIGiolabdQeakbqab0GaeyyeIuoakmaaCaaaleqabaGaeGOmaidaaOGaeyOKH4QagiyBa0MaeiyAaKMaeiOBa4gaaaaa@B3C8@

where *x*._*j *_denotes the average expression of genes on the diagnostic array *j*, *J *is the set of all samples, *I*_*d *_is the set of all genes on the diagnostic microarray and *x*.. the average gene expression over all diagnostic microarrays. This is done using a greedy forward selection, which is summarized in pseudo code. In contrast to the methods above, the normalization is now done using both signature and normalization genes. The strategy here is not to find genes that are not affected by expression difference between the two disease groups, but genes that compensate this effect. For example, if the signature genes are all up-regulated in group A, the goal is to compensate for this effect by choosing genes which are down regulated. This method does not distinguish between the discriminating genes and the genes for normalization any more. The normalization genes are now themselves differentially expressed and can hence be included into the signature.

In the absence of experimental artifacts the summed up expression levels for each sample should be constant. In this way, these genes allow us to distinguish between differential expression and experimental artifacts. Similar to the first two methods, there is again a generalization problem. We balance the signature on the training set. Its normalization performance for the diagnostic microarray however depends on how well the balance between up- and down-regulated genes generalizes to new data.

### Normalization of small diagnostic microarrays

Normalization of small diagnostic microarrays was done by subtracting the sample wise mean of the normalization genes from all genes. Let *x*_*ij *_be the expression of gene *i *in patient *j*. Let *I*_*n *_be the set of normalization genes, and *p*_*n *_= |*I*_*n*_| the number of normalization genes. For all normalization genes the sample wise mean *V*_*j *_was calculated: νj=∑i∈Inxijpn
 MathType@MTEF@5@5@+=feaafiart1ev1aaatCvAUfKttLearuWrP9MDH5MBPbIqV92AaeXatLxBI9gBaebbnrfifHhDYfgasaacH8akY=wiFfYdH8Gipec8Eeeu0xXdbba9frFj0=OqFfea0dXdd9vqai=hGuQ8kuc9pgc9s8qqaq=dirpe0xb9q8qiLsFr0=vr0=vr0dc8meaabaqaciaacaGaaeqabaqabeGadaaakeaaiiGacqWF9oGBdaWgaaWcbaGaemOAaOgabeaakiabg2da9maaqababaWaaSaaaeaacqWG4baEdaWgaaWcbaGaemyAaKMaemOAaOgabeaaaOqaaiabdchaWnaaBaaaleaacqWGUbGBaeqaaaaaaeaacqWGPbqAcqGHiiIZcqWGjbqsdaWgaaadbaGaemOBa4gabeaaaSqab0GaeyyeIuoaaaa@3FE4@. Normalization was then done by subtracting *V*_*j *_from all genes resulting in normalized data *y*_*ij*_: *y*_*ij *_= *x*_*ij *_- *v*_*j*_. For the balanced signature *I*_*n *_included all genes and therefore *V*_*j *_= *x*_.*j*_

## Authors' contributions

JJ performed the simulation and data analysis, and contributed to the design of the study and the writing of the manuscript. RS contributed to the design of the study and the writing of the manuscript. All authors read and approved the final manuscript.

## Greedy forward selection

**Let**: *J *= *J*_*A *_∪ *J*_*B*_, be all samples in group A and B, |*J*| is the number of all samples

*I*_*l*_, be the set of all genes on the large microarray

*I*_*s*_, be the set of given genes of the diagnostic signature

*I*_*n *_= {}, be the initially empty set of normalization genes

**for ***k *= 1... *p*_*n *_(for each normalization gene)

*I*_*d *_= *I*_s _∪ *I*_*n*_

**for ***g *∈ *I*_*l*_\*I*_*d *_(for each gene on the large microarray not yet used on the diagnostic microarray) calculate vg=∑j∈J(∑i∈Id∪g(xij−∑j∈Jxij|J|))2
 MathType@MTEF@5@5@+=feaafiart1ev1aaatCvAUfKttLearuWrP9MDH5MBPbIqV92AaeXatLxBI9gBaebbnrfifHhDYfgasaacH8akY=wiFfYdH8Gipec8Eeeu0xXdbba9frFj0=OqFfea0dXdd9vqai=hGuQ8kuc9pgc9s8qqaq=dirpe0xb9q8qiLsFr0=vr0=vr0dc8meaabaqaciaacaGaaeqabaqabeGadaaakeaacqWG2bGDdaWgaaWcbaGaem4zaCgabeaakiabg2da9maaqababaWaaeWaceaadaaeqaqaamaabmGabaGaemiEaG3aaSbaaSqaaiabdMgaPjabdQgaQbqabaGccqGHsisldaWcaaqaamaaqababaGaemiEaG3aaSbaaSqaaiabdMgaPjabdQgaQbqabaaabaGaemOAaOMaeyicI4SaemOsaOeabeqdcqGHris5aaGcbaWaaqWaceaacqWGkbGsaiaawEa7caGLiWoaaaaacaGLOaGaayzkaaaaleaacqWGPbqAcqGHiiIZcqWGjbqsdaWgaaadbaGaemizaqgabeaaliabgQIiilabdEgaNbqab0GaeyyeIuoaaOGaayjkaiaawMcaaaWcbaGaemOAaOMaeyicI4SaemOsaOeabeqdcqGHris5aOWaaWbaaSqabeaacqaIYaGmaaaaaa@591D@

*I*_*n *_= *I*_*n *_∪ argmin_*g *_*v*_*g*_

Pseudo code for greedy forward selection of balancing genes

## Figures and Tables

**Figure 5:**
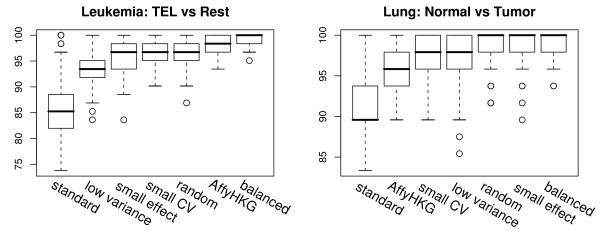
**Classification accuracy using different normalization methods**. Cross validation results of predictive performance of the same diagnostic signature used with different normalization strategies for diagnostic microarrays. The left plot shows classification accuracies for distinguishing TEL-AML1 from other groups in leukemia (*p*_*s *_= *p*_*n *_= 5). The right plot shows classification accuracies for distinguishing normal from adenocarcinomas in lung (*p*_*s *_= *p*_*n *_= 3). The boxplots are sorted by increasing median accuracy. When they have the same median the mean was used for sorting.
